# P-707. Relative Effectiveness of Cell-Based Influenza Vaccines versus Egg-Based Influenza Vaccines: A Review of Test-Confirmed and Clinical Diagnosis-Based Outcomes

**DOI:** 10.1093/ofid/ofae631.903

**Published:** 2025-01-29

**Authors:** Mahrukh Imran, Edin Mifsud, Mendel Haag, Ian McGovern

**Affiliations:** CSL Seqirus, Toronto, Ontario, Canada; CSL Seqirus, Toronto, Ontario, Canada; CSL Seqirus, Toronto, Ontario, Canada; CSL Seqirus, Toronto, Ontario, Canada

## Abstract

**Background:**

Cell-based influenza vaccine viruses may more closely match recommended vaccine strains than egg-based options. A previous systematic literature review (SLR) and meta-analysis evaluated the relative effectiveness (rVE) of cell-based quadrivalent influenza vaccine (QIVc) versus standard-dose, egg-based quadrivalent or trivalent influenza vaccines (QIVe/TIVe) in preventing either clinically diagnosed or test-confirmed influenza. We sought to build on that prior evidence and evaluate the rVE of QIVc versus QIVe/TIVe by separately analyzing test-confirmed and clinically diagnosed influenza.

**Methods:**

Studies reporting on the rVE of QIVc vs QIVe/TIVe among persons aged 4–64 years were identified from a prior SLR (PROSPERO CRD42020160851) that included studies published between 01-January-2016 and 25-February-2022. Additionally, a scoping review was conducted to identify additional studies published between 25-February-2022 and 01-March-2024. A DerSimonian and Laird random effects model was applied for the meta-analyses.

**Results:**

The SLR included 10 relevant publications (n=3 test-confirmed; n=7 clinical diagnosis) spanning three influenza seasons from 2017–2020. An additional test-confirmed study covering the 2017–2020 influenza seasons was identified from the scoping review. Among persons aged 4–64 years, the pooled rVE demonstrated a consistent benefit of QIVc for both outcome types, with estimates of 11.9% (95% CI, 3.0%–20%; n studies=3) for 2017–2018, 11.8% (4.2%–19.4%; n=2) for 2018–2019, and 10.0% (2.7%–16.7%; n=1) for 2019–2020 in preventing test-confirmed influenza and 18.7% (8.7%–27.6%; n=2) for 2017–2018, 5.9% (4.3%–7.5%; n=2) for 2018–2019, and 10.1% (6.1%–14.0%; n=2) for 2019–2020 in preventing clinically diagnosed influenza.

**Conclusion:**

For persons aged 4–64 years, QIVc was consistently more effective than QIVe/TIVe over the three influenza seasons for both test-confirmed influenza and clinically diagnosed influenza. The results do not suggest systematic over or underestimation of rVE for prevention of clinically diagnosed compared to test-confirmed influenza.

**Disclosures:**

**Mahrukh Imran, MScPH**, CSL Seqirus: Employee **Edin Mifsud, PhD**, CSL Seqirus: Employee|CSL Seqirus: Stocks/Bonds (Private Company) **Mendel Haag, PhD, PharmD**, CSL Seqirus: Employment|CSL Seqirus: Stocks/Bonds (Public Company) **Ian McGovern, MPH**, CSL Seqirus: Employee|CSL Seqirus: Stocks/Bonds (Public Company)

